# The Effect of Centrifugal Force in Quantification of Colorectal Cancer-Related mRNA in Plasma Using Targeted Sequencing

**DOI:** 10.3389/fgene.2018.00165

**Published:** 2018-05-15

**Authors:** Vivian Weiwen Xue, Simon Siu Man Ng, Wing Wa Leung, Brigette Buig Yue Ma, William Chi Shing Cho, Thomas Chi Chuen Au, Allen Chi Shing Yu, Hin Fung Andy Tsang, Sze Chuen Cesar Wong

**Affiliations:** ^1^Department of Health Technology and Informatics, Faculty of Health and Social Sciences, Hong Kong Polytechnic University, Kowloon, Hong Kong; ^2^Department of Surgery, Faculty of Medicine, The Chinese University of Hong Kong, Shatin, Hong Kong; ^3^State Key Laboratory in Oncology in South China, Sir YK Pao Centre for Cancer, Department of Clinical Oncology, Hong Kong Cancer Institute and Li Ka Shing Institute of Health Sciences, The Chinese University of Hong Kong, Shatin, Hong Kong; ^4^Department of Clinical Oncology, Queen Elizabeth Hospital, Kowloon, Hong Kong; ^5^Department of Computer Science, University of Oxford, Oxford, United Kingdom

**Keywords:** centrifugal force, targeted sequencing, plasma mRNA, colorectal cancer, gene expression

## Abstract

In our previous study, we detected the effects of centrifugal forces on plasma RNA quantification by quantitative reverse transcription PCR. The aims of this study were to perform targeted mRNA sequencing and data analysis in healthy donors' plasma prepared by two centrifugation protocols and to investigate the effects of centrifugal forces on plasma mRNA quality and quantity. Targeted mRNA sequencing was performed using a custom panel with 108 colorectal cancer-related genes in 18 healthy donors' plasma that prepared by (1) 3,500 g for 10 min at 4°C and (2) 1,600 g for 10 min at 4°C followed by 16,000 g for 10 min at 4°C. Results showed that plasma ribosomal RNA was detected in 16/18 (88.9%) 3,500 g and 6/18 (33.3%) 1,600 g followed by 16,000 g centrifuged plasma. For targeted sequencing, 75/108 (69.4%) and 86/108 (79.6%) genes were detected in 3,500 and 1,600 g followed by 16,000 g, respectively, while 16/108 (14.8%) genes were not detected in both centrifugations. Detailed analysis showed that 2 of 108 (1.85%) genes showed lower expressions in 3,500 g than in 1,600 g followed by 16,000 g. The median expressions of genes in 3,500 g were positively correlated with the expressions in 1,600 g followed by 16,000 g (*R*^2^ = 0.9471, *P* < 0.0001, Spearman rank correlation). Meanwhile, plasma samples were not distinctively clustered based on centrifugal forces according to hierarchical clustering. Targeted mRNA sequencing and subsequent data analysis were performed in this study to investigate the effects of two different centrifugal forces that are commonly used in plasma collection. Our targeted sequencing results help to understand the centrifugal force effects on plasma mRNA, and these findings show that the centrifugation protocol for plasma mRNA research using targeted sequencing can be standardized which facilitates multicenter studies for comparison and quality assurance in the future.

## Introduction

Colorectal cancer (CRC) is one of the most serious health issues worldwide (Brenner et al., 2014). The survival rate of primary CRC is significantly higher than the rate of advanced cancer, which means that a more effective cancer screening is helpful in CRC prevention (Levin et al., 2008). Currently recommended annual screening for people age ≥ 50 years is Guaiac-based fecal occult blood test (gFOBT) or fecal immunochemical test (FIT) (Smith et al., 2017). Although they are economical and non-invasive tests, gFOBT is not specific enough due to false-positive detection from hemorrhoids and ulcers, and both gFOBT and FIT are not sensitive in neoplasm detection (Brenner and Tao, 2013; Widlak et al., 2017). The insufficient sensitivity of current screening for non-invasive and early detection of CRC urges a more effective non-invasive detection based on liquid biopsy such as plasma. Plasma mRNA has been used as diagnostic and prognostic tumor markers in various cancers (García et al., 2007). The detection of plasma mRNA is non-invasive and flexible, which is beneficial to cancer patients' follow-up after surgeries or adjuvant therapies, and it has potential to monitor cancer recurrence as well (Wong et al., 2004b; Stein et al., 2011). However, mRNA has a low abundance, and it is fragmented in plasma (Savelyeva et al., 2017). Moreover, different blood processing protocols, such as filtering and different centrifugal forces used in plasma preparation, may result in different quantification of plasma mRNA (Ng et al., 2002; El-Hefnawy et al., 2004; Wong et al., 2007). In a previous study, an additional centrifugation in 1,300 g for 10 min after a routine centrifugation in 1,800 g for 10 min was found to decrease plasma RNA concentration over 20 times (El-Hefnawy et al., 2004). Moreover, our earlier work showed that mRNA quantity was significantly different in metastatic CRC patients' plasma samples prepared by two centrifugations, 800 g for 8 min at 4°C and 4,500 g for 8 min at 4°C, which detected *CTNNB1, SELP, KRT20*, and *GAPDH* mRNA using quantitative reverse transcription PCR (RT-qPCR) (Wong et al., 2007). Those results demonstrated that different centrifugal forces could lead to different quantities of mRNA in plasma samples from CRC patients. This artifact alerts us the importance to standardize the centrifugal protocol for plasma mRNA analysis. Otherwise, the data obtained cannot be interpreted and compared with other studies.

With the prevalence of next-generation sequencing, RNA deep sequencing has been used as an approach for transcriptome profiling in plasma samples (Wang et al., 2014; Shih et al., 2015). However, there is no standardized protocol of plasma preparation or explicit descriptions of centrifugal forces effects on plasma mRNA, which has crucial impacts on reproducibility of applications and multicenter researches. Some studies used plasma samples collected from centrifugal forces, such as 1,400 or 1,600 g, to study microRNAs (miRNAs) and mRNAs cancer markers using RNA deep sequencing, respectively (Wang et al., 2014; Shih et al., 2015). On the other hand, some researchers used a higher centrifugal forces to profile plasma extracellular RNAs using small RNA deep sequencing (Freedman et al., 2016; Danielson et al., 2017). In one study, plasma samples were prepared in 2,500 g for 22 min at 4°C with an additional centrifugation in 8,000 g for 5 min after plasma thawing prior to RNA extraction (Freedman et al., 2016). In another study, plasma samples were prepared in 1,000 g for 10 min at room temperature with an additional centrifugation in 2,000 g after plasma thawing prior to RNA extraction (Danielson et al., 2017). Up to now, no study has reported the effect of centrifugal force on plasma mRNA based on RNA sequencing data. Therefore, we aim to examine the effect of centrifugal force on plasma mRNA quantity and quality that may be important for cancer detection and monitoring.

In this study, we examined a panel of CRC-related mRNA in healthy donors' (HDs) plasma prepared by two commonly used centrifugations (1) 3,500 g for 10 min at 4°C and (2) 1,600 g for 10 min at 4°C followed by 16,000 g for 10 min at 4°C. CRC-related mRNA expression was detected using targeted deep sequencing. Subsequently, differential expression and correlation of expression in two centrifugal forces were analyzed. The information obtained from this study will be helpful for us to understand the centrifugal force effects on the expression level of CRC-related mRNAs in plasma samples, which could facilitate us to develop a standardized and effective protocol of CRC biomarker detection using targeted mRNA sequencing in plasma samples.

## Materials and methods

### Healthy donors recruitment and plasma collection

Eighteen HDs were recruited in this study. For each donor, 15 ml peripheral blood was collected in K3 EDTA tubes (Greiner Bio-one, Austria) and divided to two parts evenly. One portion was centrifuged for 3,500 g, 10 min at 4°C, and 3.2 ml plasma was collected and preserved by 9.6 ml Trizol LS Reagent (Thermo Fisher Scientific, USA) before storage at −80°C. Another portion was centrifuged for 1,600 g, 10 min at 4°C followed by 16,000 g for 10 min at 4°C, and 3.2 ml plasma was collected and preserved in the same way. Microfuge 22R Centrifuge and F301.5 rotor (Beckman Coulter) were used for centrifugation in plasma preparation. Blood processing was done within 4 h after blood draw. All donors were recruited with written informed consent. The study was approved by the Joint Chinese University of Hong Kong and New Territories Easter Cluster Clinical Research Ethics Committee (CREC-2014.224).

### RNA extraction and purification

For each sample, 3.2 ml plasma was used for RNA extraction using our established protocol (Wong et al., 2004a,b). In brief, the aqueous layer with RNA was separated after adding chloroform (Sigma-Aldrich, USA) followed by centrifugation for 12,000 g, 15 min at 4°C. Then, 0.54 volume of absolute ethanol (Sigma-Aldrich, USA) was added to the aqueous layer to achieve appropriate binding conditions. The mixture was purified using RNeasy Mini Kit (Qiagen, Germany) (Wong et al., 2004a,b). Subsequently, DNase digestion using TURBO DNA-free Kit (Invitrogen, Lithuania) was performed, and the DNA-free RNA was concentrated using RNeasy MinElute Cleanup Kit (Qiagen, Germany) according to the manufacturer's instructions (Tsui et al., 2014). Plasma total RNA was eluted in 14 μl RNase-free water, and it was stored at −80°C until use. The quality and quantity of RNA was detected using Agilent RNA 6000 Pico Kit (Agilent Technologies, Lithuania) on 2100 Bioanalyzer. RNA integrity number (RIN) and the percentage of RNA fragments > 200 nt (DV_200_) were detected as the quality indicators. RIN is a standardized value to describe RNA quality, which has considered the 28S/18S ratio and other features from electrophoretic RNA separation results (Schroeder et al., 2006). DV_200_ is a parameter to evaluate the length distribution of fragmented RNA (Landolt et al., 2016).

### Sequencing library preparation and data analysis

Sequencing library was prepared using a custom designed TruSeq Targeted RNA Expression Kit (Illumina, USA), which was used to examine a panel of 108 CRC-related genes including 93 Wnt-signaling genes, existing CRC markers from literatures and a control gene (Supplementary Table 1). The cDNA libraries were synthesized using 5 μl extracted plasma RNA, which was equivalent to about 670 pg RNA per sample, and the preparation of sequencing libraries was according to the manufacturer's instructions with slight modifications, including (1) 2-fold diluted adapters to amplify libraries and (2) two times of clean-up for PCR products using AMPure XP beads (Beckman Coulter, USA) (Tsui et al., 2014). The quality and quantity of prepared cDNA libraries were checked by Agilent High Sensitivity DNA Kit (Agilent Technologies, Lithuania) and qPCR, respectively. FastStart Universal SYBR Green Master (Roche, Germany) was used in quantification. Primers with 5′-AATGATACGGCGACCACCGAGAT-3′ and 5′-CAAGCAGAAGACGGCATACGA-3′ matched sequences within adapters were used. Illumina format DNA standard (Qiagen, Germany) was prepared by a serial dilution to achieve the standard curve for absolute quantification. The pooled sequencing library with 5% PhiX control (Illumina) was sequenced for single-end 51 bp length on MiSeq System using MiSeq Reagent Kit v3 (Illumina, Singapore). The targeted RNA sequencing data were available in Sequence Read Archive (SRA) database (SRP125573).

Data analysis for targeted mRNA sequencing included two parts. The primary analysis was performed on MiSeq reporter. After base calling, FASTQ files of sequences with high sequencing quality were aligned to hg19 reference genome based on custom designed regions. Raw aligned replicate counts of each gene for each sample were output. Counts per million (CPM) of genes were calculated as normalized expression for correcting biases due to library sizes. Let the raw count of gene *i* in a sample *j* is *C*_*ij*_, with *i* = 1 to *n* and *j* = 1 to *m*. The calculation of *CPM*_*ij*_ is below (Rau et al., 2013; Law et al., 2014):

$$CPM_{ij}=\frac{C_{ij}}{\sum_{i=1}^{n}C_{ij}}\times 10^{6}$$

The normalized expression of gene was shown using log_2_ scale as *log*_2_ *(CPM*+*1*) to avoid log transformation for zero CPM (Law et al., 2014; Tsui et al., 2014). The secondary analysis was performed as below pipeline: a non-specific filter as “keep the gene if it has > 1 CPM in ≥ 18 plasma samples” was used to remove uninformative signals and increase detection power without dependency on centrifugal force labels, which generally excluded low-abundance genes and reduced dispersion (Bourgon et al., 2010; Robinson et al., 2010; Rau et al., 2013). After the filtering, DESeq2 was used to estimate dispersion and detect differential expression using paired sample test for the remaining genes (Love et al., 2014). A cutoff of fold change > 4 and adjusted *P* value < 0.05 was used to identify significant differences. Adjusted *P* value was calculated based on Benjamini-Hochberg correction (Benjamini and Hochberg, 1995).

### Statistical analysis

Statistical analysis was performed by Wilcoxon matched-pairs signed rank test and Spearman correlation in Prism 5. *P* < 0.05 was regarded as significant difference and significant correlation, respectively.

## Results

### Plasma RNA quality and quantity from two centrifugal forces

**Figure 1:**
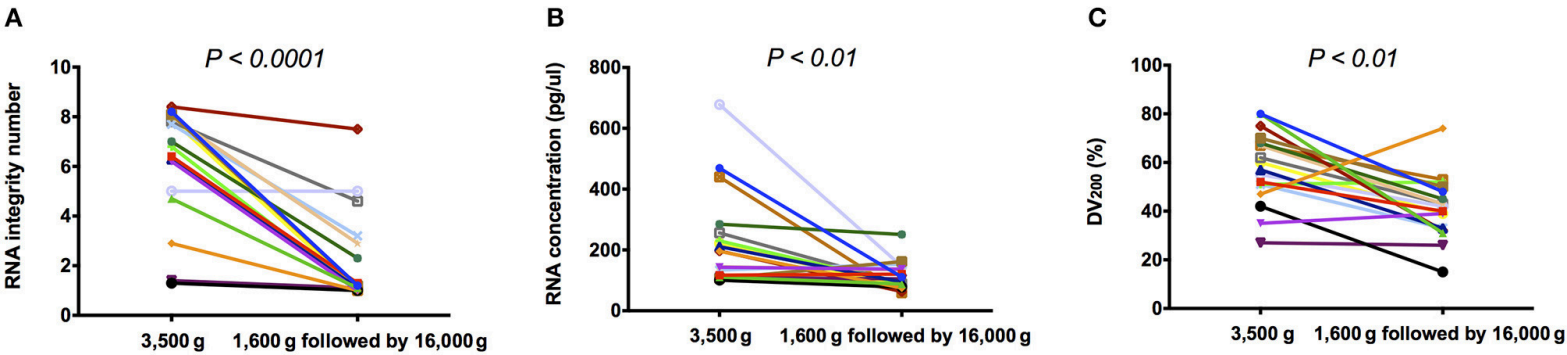
The quality and quantity of plasma RNA. **(A)** RIN; **(B)** RNA concentration; and **(C)** DV_200_ of extracted plasma total RNA in 3,500 g were significantly higher than those in 1,600 g followed by 16,000 g (*P* < 0.05, Wilcoxon matched-pairs signed rank test).

Using Bioanalyzer, 18S and 28S rRNAs were detected in 16/18 (88.9%) 3,500 g centrifuged plasma and 6/18 (33.3%) 1,600 g followed by 16,000 g centrifuged plasma, respectively (Supplementary Figure 1). Besides, RIN was detected with median of 6.90 (range: 1.3–8.4) and 1.15 (range: 1–7.5) in 3,500 and 1,600 g followed by 16,000 g centrifuged plasma, respectively. RIN in 3,500 g was significantly higher than those in 1,600 g followed by 16,000 g (Figure 1A, *P* < 0.0001, Wilcoxon matched-pairs signed rank test). RNA concentration was detected with median concentration of 196.5 (range: 101–678) and 100.0 (range: 59–251) pg/μl in 3,500 and 1,600 g followed by 16,000 g centrifuged plasma, respectively. RNA concentration in 3,500 g was significantly higher than those in 1,600 g followed by 16,000 g (Figure 1B, *P* < 0.01, Wilcoxon matched-pairs signed rank test). DV_200_ was detected with median percentage of 58.5 (range: 27–80) and 41.0 (range: 15–74) in 3,500 and 1,600 g followed by 16,000 g centrifuged plasma, respectively. DV_200_ in 3,500 g was significantly higher than those in 1,600 g followed by 16,000 g (Figure 1C, *P* < 0.01, Wilcoxon matched-pairs signed rank test).

### Summary of plasma mRNA targeted sequencing

Overall, the number of total raw reads in targeted sequencing on MiSeq is 39.0 million with at least 93% ≥ Q30. Among them, about 32.9 million reads were high quality, and 20.9% of them were aligned to the targeted regions in human genome hg19.

Detection of the 108 CRC-related genes was summarized in Table 1. Sixteen (14.8%) of 108 genes were undetectable in both centrifugations. Besides, 6 genes could only be detected in 3,500 g but not in 1,600 g followed by 16,000 g centrifuged plasma, while 17 genes could only be detected in 1,600 g followed by 16,000 g but not in 3,500 g centrifuged plasma. Details of the genes detected only in one centrifugal force condition were listed in Table 2. These 23 genes were detected in ≤ 5 plasma samples, and the majority of them (73.9%) were low-abundance (raw counts ≤ 10 counts).

**Table 1:** The summary of detection of genes in two centrifugal forces.

	**3,500 g**	**1,600 g followed by 16,000 g**
**Genes (*****n*** = **108)**		
Detectable	75 (69.4%)	86 (79.6%)
In both centrifugations	69 (63.8%)	69 (63.8%)
Only in this centrifugation	6 (5.6%)	17 (15.8%)
Undetectable	33 (30.6%)	22 (20.4%)
In both centrifugations	16 (14.8%)	16 (14.8%)
Only in this centrifugation	17 (15.8%)	6 (5.6%)

**Table 2:** The expression of genes detected only in one of two centrifugal forces.

**Centrifugation**	**Gene**	**Detectable**	**Raw counts in each detectable sample (count)**	**Normalized expression in each detected sample (CPM)**	**Range of normalized expression (CPM)**
3,500 g	AXIN1	1/18	1	43.6	43.6
	BIRC5	5/18	2, 1, 2, 4, 1	2.7, 0.5, 191.6, 691.7, 10.7	0.5–691.7
	PORCN	1/18	1	0.5	0.5
	TBP	1/18	844	24825.0	24825.0
	WNT7B	2/18	8, 1	7.9, 10.7	7.9–10.7
	WNT10A	1/18	1	1.3	1.3
1,600 g followed by 16,000 g	AXIN2	2/18	1, 1	11.8, 34.6	11.8–34.6
	FOSL1	1/18	1	524.4	524.4
	FRZB	1/18	1	524.4	524.4
	FZD9	1/18	56	503.0	503.0
	IL6	5/18	1, 1, 1, 3, 25	7.8, 44.0, 9.0, 1573.2, 242.4	7.8–1573.2
	LRP6	2/18	2, 6	1048.8, 58.2	58.2–1048.8
	NANOG	1/18	1	9.7	9.7
	PRICKLE1	2/18	2, 1	1048.8, 9.7	9.7–1048.8
	SFRP1	3/18	4, 23, 18	984.0, 12060.8, 174.6	174.6–12060.8
	SFRP2	4/18	6, 1, 55, 10	2051.3, 11.8, 28841.1, 97.0	11.8–28841.1
	SFRP4	2/18	1, 15	44.0, 7865.8	44.0–7865.8
	SOX17	1/18	3	29.1	29.1
	TCF7L1	1/18	1	9.7	9.7
	WIF1	1/18	3	3.6	3.6
	WNT2B	1/18	1	1.2	1.2
	WNT4	2/18	1, 1	3.4, 9.7	3.4–9.7
	WNT5B	1/18	1	9.0	9.0

### Expression filtering and gene expression levels in two centrifugal forces

**Figure 2:**
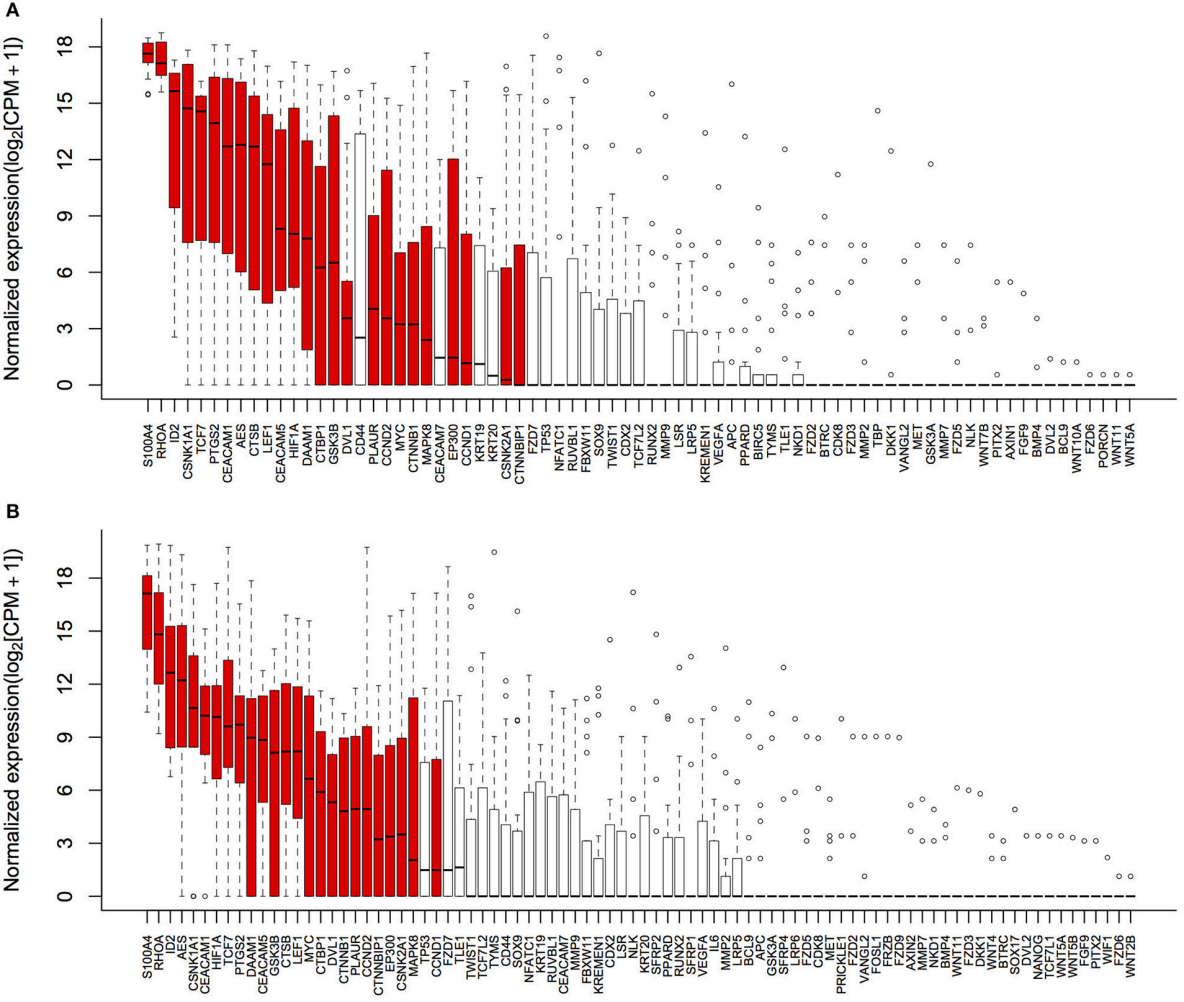
Gene expressions in two centrifugal forces. Normalized expressions (log_2_ scale) of genes were calculated as *log*_2_ *(CPM*+*1)* to avoid log transformation for zero CPM. **(A)** 75 genes were detectable in 3,500 g; and **(B)** 86 genes were detectable in 1,600 g followed by 16,000 g. Box and whisker plots showed the interquartile range of log-transformed normalized expressions and outliers, and 25 passed filter genes were highlighted by red color.

**Figure 3:**
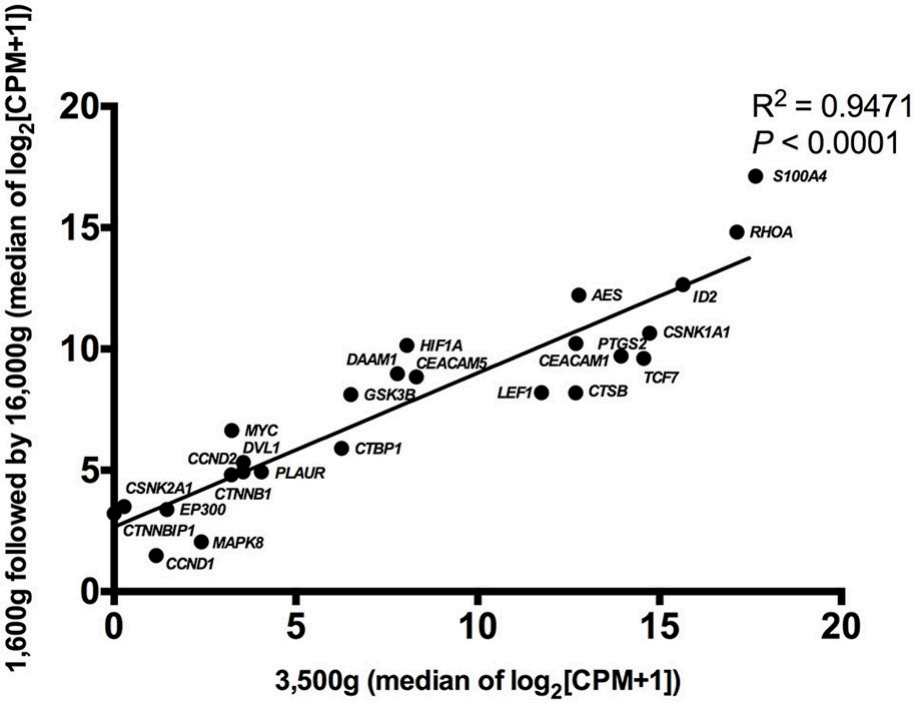
The correlation of gene expressions in two centrifugal forces. The median normalized expressions (log_2_ scale) of 25 passed filter genes were correlative between 3,500 and 1,600 g followed by 16,000 g conditions (*R*^2^ = 0.9471, *P* < 0.0001, Spearman rank correlation).

Detectable genes in 3,500 g (Figure 2A) and 1,600 g followed by 16,000 g (Figure 2B) were 75 genes and 86 genes, respectively. Based on the normalized expression levels (CPM), 25 of 108 (23.1%) genes that had the higher expression compared to the majority of other genes in both centrifugations passed the filter (> 1 CPM in ≥ 18 plasma samples), and they were highlighted by red color in Figure 2. Only these 25 genes were included in the following differential expression analysis. The median expressions of those genes in 3,500 g were positively correlated with the expressions in 1,600 g followed by 16,000 g (Figure 3, *R*^2^ = 0.9471, *P* < 0.0001, Spearman rank correlation). On the other hand, 83 genes were filtered out because of their low sequencing coverage, and they were not included in downstream differential expression analysis.

### Differential gene expression in two centrifugal forces

The differential expression analysis and the fold-change estimation of 25 passed filter genes were performed by DESeq2. Results were listed in Table 3, and genes with significant difference in expression were highlighted in bold. Among them, MYC proto-oncogene (*MYC*) and hypoxia inducible factor 1 alpha subunit (*HIF1A*) showed significantly lower expressions in 3,500 g compared with in 1,600 g followed by 16,000 g (16.67-fold with adjusted *P* < 0.005 and 5.56-fold with adjusted *P* < 0.05, respectively). In detail, *MYC* was detected in 11/18 (61.1%) plasma samples in both centrifugations with the median normalized expression of 9.2 (range: 0.0–30443.0) and 98.9 (range: 0.0–48931.0) CPM in 3,500 g and 1,600 g followed by 16,000 g, respectively. *HIF1A* was detected in 15/18 (83.3%) plasma samples in both centrifugations with the median normalized expression of 266.0 (range: 0.0–149880.3) and 1135.8 (range: 0.0–212939.9) CPM in 3,500 and 1,600 g followed by 16,000 g, respectively.

**Table 3:** The differential gene expression in two centrifugal forces analyzed by DESeq2 (3,500 vs. 1,600 g followed by 16,000 g).

**Gene ID**	**Chr**	**Start**	**Stop**	**Fold change**	***P* Value**	**Adjusted *P* Value**
***MYC***	**8q24.21**	**128748825**	**128750534**	**0.06**	**0.0001**	**0.0025**
***HIF1A***	**14q23.2**	**62187260**	**62188264**	**0.18**	**0.0037**	**0.0457**
*CCND1*	11q13.3	69456247	69457833	0.15	0.0077	0.0645
*AES*	19p13.3	3061188	3057697	0.26	0.0159	0.0993
*CCND2*	12p13.32	4385360	4387954	0.25	0.1028	0.3402
*CEACAM5*	19q13.2	42212680	42213625	0.39	0.1225	0.3402
*CTNNB1*	3p22.1	41241134	41265540	0.33	0.1154	0.3402
*DAAM1*	14q23.1	59730348	59757978	0.35	0.0961	0.3402
*PLAUR*	19q13.31	44171775	44169568	0.30	0.1138	0.3402
*DVL1*	1p36.33	1284308	1278111	0.38	0.1631	0.4078
*CSNK2A1*	20p13	485804	480536	0.44	0.2674	0.4489
*CTNNBIP1*	1p36.22	9937997	9932118	0.40	0.2694	0.4489
*MAPK8*	10q11.22	49609793	49612930	0.39	0.2324	0.4489
*PTGS2*	1q31.1	186648486	186648291	1.96	0.2334	0.4489
*S100A4*	1q21.3	153517158	153516365	0.60	0.2344	0.4489
*CTSB*	8p23.1	11710868	11710176	1.77	0.3313	0.5019
*GSK3B*	3q13.33	119812228	119721043	0.54	0.3413	0.5019
*EP300*	22q13.2	41489064	41513219	0.55	0.4247	0.5898
*CTBP1*	4p16.3	1232001	1222088	0.63	0.5014	0.6597
*ID2*	2p25.1	8822615	8823016	0.72	0.5326	0.6657
*CSNK1A1*	5q32	148930433	148929706	1.23	0.7224	0.8209
*LEF1*	4q25	109086283	109084819	0.79	0.7068	0.8209
*CEACAM1*	19q13.2	43031227	43026331	1.09	0.8773	0.9139
*TCF7*	5q31.1	133473825	133474683	0.89	0.8506	0.9139
*RHOA*	3p21.31	49412899	49405938	1.02	0.9613	0.9613

**Figure 4:**
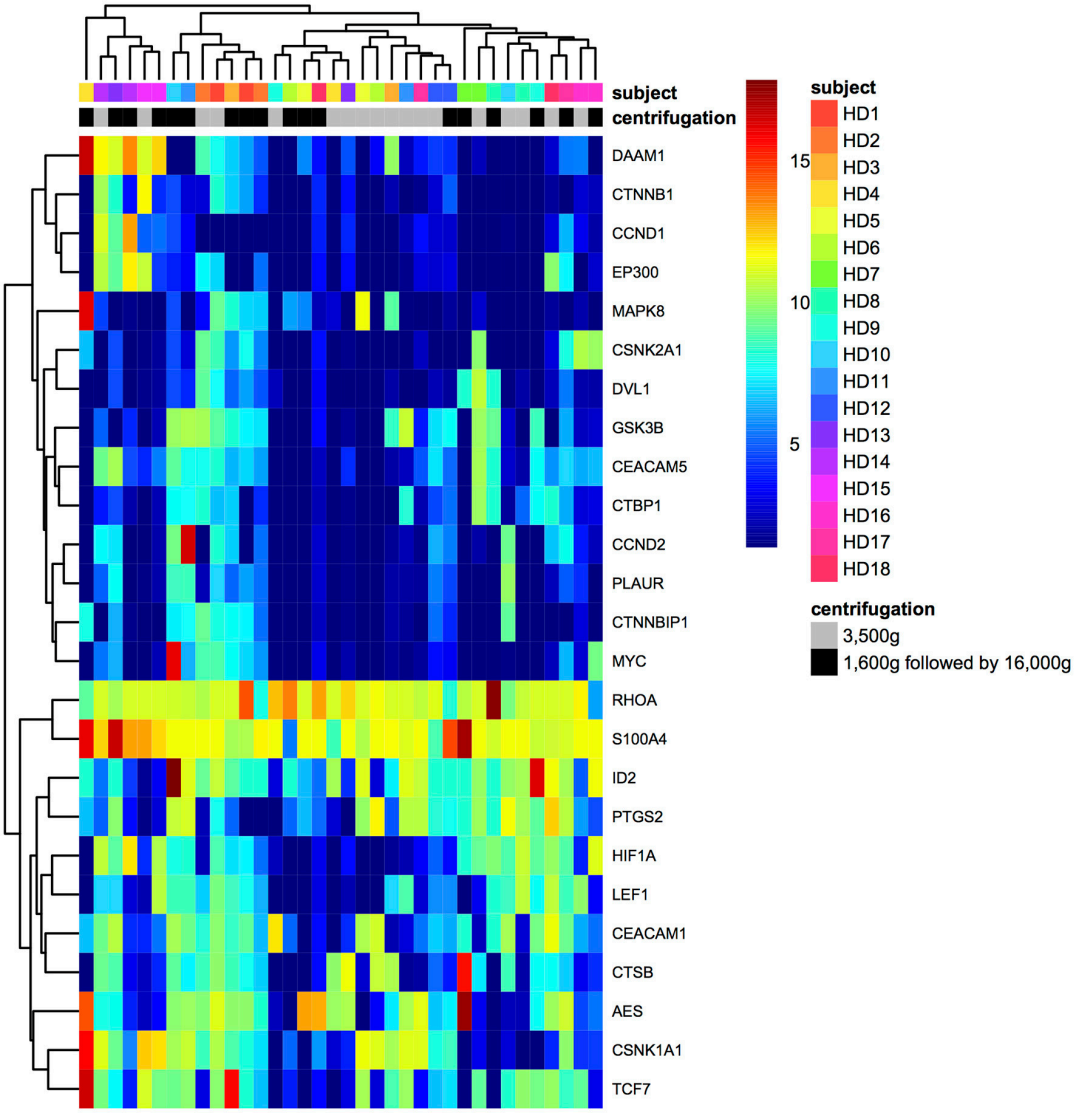
The hierarchical clustering for samples and gene expressions.

The hierarchical clustering for samples and gene expressions in different centrifugations was achieved by complete linkage of Euclidean distances (Figure 4). Plasma samples were not distinctively clustered based on centrifugal forces, which indicated 3,500 and 1,600 g followed by 16,000 g centrifugations could not cause distinguished differential expression to the panel of CRC-related genes in HDs' plasma samples.

## Discussion

Plasma RNA sequencing has been used to investigate circulating cancer markers. However, majority of previous studies focused on profiling miRNA markers, because miRNAs were relative stable in human plasma (Mitchell et al., 2008; Wang et al., 2014). For plasma mRNA, we previously reported that its quantification could be affected by different centrifugal forces based on RT-qPCR results (Wong et al., 2007). Therefore, it is important to explicate centrifugal force effects based on RNA deep sequencing data before examining plasma mRNA using RNA deep sequencing technologies. Here, we provided important information on the centrifugal force effects using a custom panel of CRC-related mRNAs in plasma samples. Basically, we found that there were 2 of 108 CRC-related genes showed differential expression in plasma samples prepared by protocols with different centrifugal forces. Besides, the results of clustering and correlation for gene expression showed that two centrifugal forces used in this study were not cause distinguished differential expression to the panel of CRC-related genes in HDs' plasma samples. This is the first study to evaluate the effects of centrifugal force on plasma mRNA quantity and quality using targeted RNA sequencing.

Through comparing plasma mRNA extracted in two centrifugal forces, which were (1) 3,500 g and (2) 1,600 g followed by 16,000 g, three important findings were observed. First, RNA concentration, integrity and the percentage of longer fragments were significantly decreased in plasma samples prepared by the high centrifugal force. Compared with the cell-free plasma samples prepared by 1,600 g followed by 16,000 g (Chiu et al., 2001), plasma samples prepared by 3,500 g include more RNAs-associated particles (Ng et al., 2002). Those particles may include cell debris, extracellular vesicles, and other particles that can combine with mRNA molecules, which contribute to the increased amount of 18S and 28S rRNAs and the corresponding increase in RNA concentration. Meanwhile, the decrease of RIN and DV_200_ in plasma samples prepared by the high centrifugal force was probably due to depleted RNAs-associated particles, which account for fewer 18S and 28S rRNAs and more fragmented RNA when plasma was subjected to the high centrifugal force, respectively. This phenomenon emphasized the effects from RNAs-associated particles to the quality and quantity of plasma total RNA in different centrifugal forces.

However, our attention is also focused on whether mRNAs could be affected by different centrifugal forces. Our second finding was that most of mRNAs in our CRC-related panel were detectable in plasma samples prepared by both centrifugations using targeted mRNA sequencing. This phenomenon is not surprising, because circulating mRNAs exist and are prevented from endogenous RNase digestion due to combination and protection from particles, for example apoptotic bodies and protein complexes (Wieczorek et al., 1985; Hasselmann et al., 2001; Ng et al., 2002). However, a majority of detectable mRNAs had low and overdispersed expression, and the similar phenomenon was found in the recent study on plasma mRNA sequencing in pregnant women (Chim et al., 2017). Besides, we found several low-abundance mRNAs (≤ 10 counts) that were only detectable in one of two centrifugations (Table 2). Most of these mRNAs were only detectable in one or two plasma samples, and they accounted for the situation that more genes were detected in 1,600 g followed by 16,000 g than in 3,500 g. It was difficult to determine which kind of particles protected these mRNAs from RNase in plasma samples. For transcripts only detected in 3,500 g-centrifuged plasma samples, their existence could be related to the presence of cell debris, extracellular vesicles and other particles, which cannot be removed effectively by the protocol with 3,500 g centrifugal force.

Filters are generally used in RNA sequencing data analysis to eliminate uninformative data points and increase detection power, and data filters were required to be chosen prudently to avoid losing type I error control in differential analysis (Bourgon et al., 2010; Rau et al., 2013). In this study, we used CPM filter for excluding mRNAs with the expression lower than filter criteria from subsequent differential expression analysis as stated in the methodology section, which was previously defined in edgeR (Robinson et al., 2010). Our third finding was that in differential expression analysis, 25 genes were detected for downstream analysis after filtering. Among them, *MYC* and *HIF1A* showed significantly lower expressions in 3,500 g than in 1,600 g followed by 16,000 g. This phenomenon implied that plasma mRNA of these two genes was hardly affected by centrifugal force effects and mainly preserved as cell-free format, which resulted in the increased relative expressions after normalization. *MYC* encodes the transcription factor c-Myc. It showed elevated expressions in different tumor cells, and it worked with the promoter regions of targeted genes (Lin et al., 2012). *HIF1A* encodes a transcription factor that responds to hypoxia through recruiting specific cyclin dependent kinase, stimulating RNA polymerase elongation and activating transcription of downstream genes (Galbraith et al., 2013). There was no previous study to describe how *MYC* and *HIF1A* mRNAs exist and are preserved as cell-free format in human plasma. The sequencing results of *MYC* and *HIF1A* expressions have been validated using RT-qPCR. Overall, those 25 genes had the high median of normalized expressions compared with other genes in both centrifugations, and their expressions in two different centrifugations were significantly correlated (Figure 3). This result demonstrated that detected gene expressions depended on the intrinsic expression levels of gene itself instead of effects from different centrifugal forces. Moreover, plasma samples were not be clustered based on the centrifugal forces used in plasma preparation, which showed that the two centrifugal forces used in this study did not lead to distinctive difference in the concentration of CRC-related mRNAs in plasma samples (Figure 4).

To conclude, we achieved targeted mRNA sequencing using a custom panel of CRC-related mRNAs in plasma samples. Our sequencing results demonstrated these plasma mRNAs were not distinctly affected by two widely different centrifugal forces. However, considering mRNA from cell debris possibly interferes disease-derived plasma mRNA quantification and the efficiency of circulating markers selection for CRC in future studies, we suggest using the protocol with 1,600 g followed by 16,000 g centrifugal force in plasma preparation, which efficiently removes cell debris from plasma and is more likely to expose disease-derived mRNA information. These findings have laid down a solid foundation in plasma RNA properties upon centrifugation for downstream RNA deep sequencing. Moreover, it is helpful for researchers to standardize their protocol so that the results generated can be compared in multicenter studies with more precision and confidence. For future works, we may use transmission electron microscopy, ultra-centrifugation and other technologies to further study which components or extracellular vesicles result in differential plasma mRNA quantification caused by centrifugal force effects.

## Author contributions

SW conceived and designed the experiments; VX performed the experiments; SW, VX, AY, and WC analyzed the data; SN, WL, BM, and WC gave invaluable comments on the subject recruitment and data interpretation; VX and SW wrote the paper; TA and HT processed the patient samples and technical work before library preparation.

### Conflict of interest statement

The authors declare that the research was conducted in the absence of any commercial or financial relationships that could be construed as a potential conflict of interest.
