# Supplementary material for: The Effect of Centrifugal Force in Quantification of Colorectal Cancer-Related mRNA in Plasma Using Targeted Sequencing
Source: Front Genet. 2018 May 15;9:165. doi: 10.3389/fgene.2018.00165 (PMC5963087; doi:10.3389/fgene.2018.00165)
Supplement: Supplementary file 2 [file Image_1.PDF]

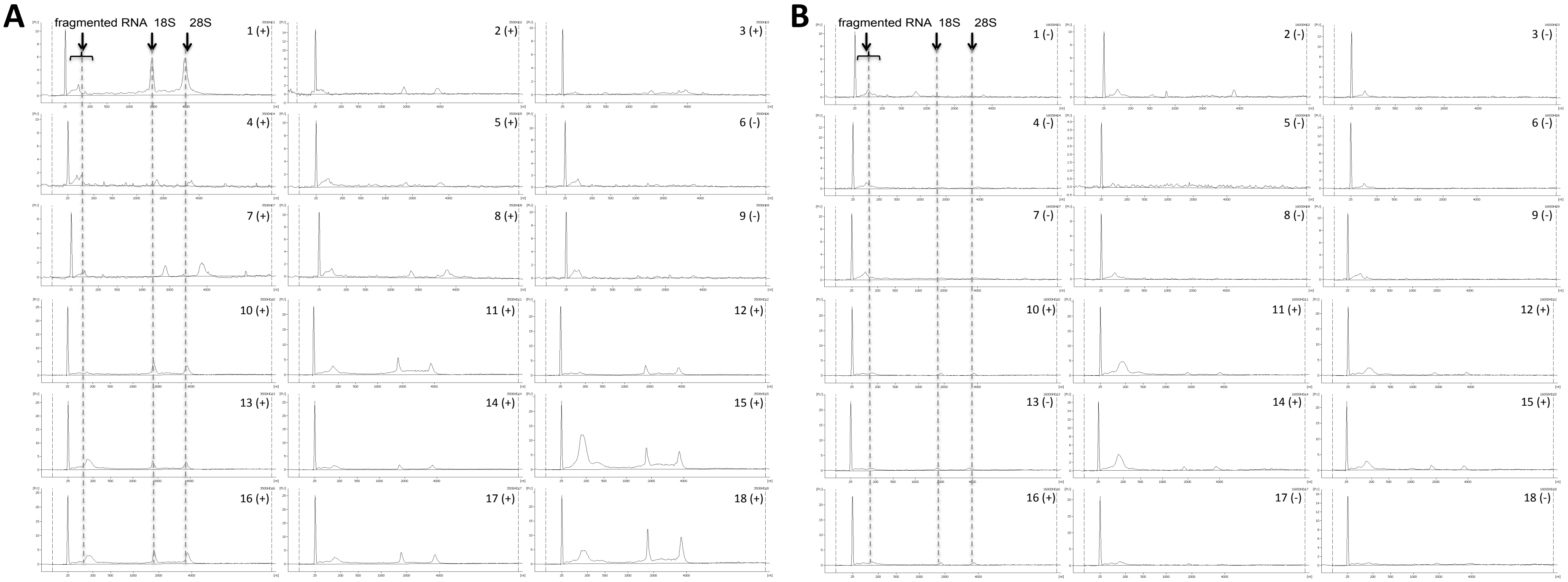

Supplementary Figure 1. The total RNA extracted from (**A**) 3,500g and (**B**) 1,600g followed by 16,000g centrifuged plasma samples were detected by Bioanalyzer. In each sample, with (+) or without (-) detectable 18S and 28S rRNAs was labeled. 18S and 28S rRNAs peaks were located around 2000 nt and 4000 nt, respectively. Extracellular mRNAs in plasma were highly fragmented with length about 200 nt or shorter. 5S rRNAs, 5.8S rRNAs, miRNAs and tRNAs ranged from 20 nt to 200 nt.
